# Privacy-Preserving Analysis of Distributed Biomedical Data: Designing Efficient and Secure Multiparty Computations Using Distributed Statistical Learning Theory

**DOI:** 10.2196/12702

**Published:** 2019-04-29

**Authors:** Fida K Dankar, Nisha Madathil, Samar K Dankar, Sabri Boughorbel

**Affiliations:** 1 United Arab Emirates University Abu Dhabi United Arab Emirates; 2 Independent Scientist Ottawa, ON Canada; 3 Sidra Medicine Doha Qatar

**Keywords:** data analytics, data aggregation, personal genetic information, patient data privacy

## Abstract

**Background:**

Biomedical research often requires large cohorts and necessitates the sharing of biomedical data with researchers around the world, which raises many privacy, ethical, and legal concerns. In the face of these concerns, privacy experts are trying to explore approaches to analyzing the distributed data while protecting its privacy. Many of these approaches are based on secure multiparty computations (SMCs). SMC is an attractive approach allowing multiple parties to collectively carry out calculations on their datasets without having to reveal their own raw data; however, it incurs heavy computation time and requires extensive communication between the involved parties.

**Objective:**

This study aimed to develop usable and efficient SMC applications that meet the needs of the potential end-users and to raise general awareness about SMC as a tool that supports data sharing.

**Methods:**

We have introduced distributed statistical computing (DSC) into the design of secure multiparty protocols, which allows us to conduct computations on each of the parties’ sites independently and then combine these computations to form 1 estimator for the collective dataset, thus limiting communication to the final step and reducing complexity. The effectiveness of our privacy-preserving model is demonstrated through a linear regression application.

**Results:**

Our secure linear regression algorithm was tested for accuracy and performance using real and synthetic datasets. The results showed no loss of accuracy (over nonsecure regression) and very good performance (20 min for 100 million records).

**Conclusions:**

We used DSC to securely calculate a linear regression model over multiple datasets. Our experiments showed very good performance (in terms of the number of records it can handle). We plan to extend our method to other estimators such as logistic regression.

## Introduction

### Background and Significance

Human genome research promises to transform health care through personalized medicine. It enables the determination of an individual’s unique molecular characteristics, which can be used to diagnose diseases, select individualized treatments (with a higher success rate), and reduce possible adverse reactions [1]. However, before this becomes a reality, more research is needed to understand the complex relationship between genome and health. Such research often requires large cohorts and necessitates the sharing of biomedical data with researchers around the world, which raises many privacy, ethical, and legal concerns.

Traditionally, researchers would strip the data from the identifying information—such as names and identity cards—and apply some privacy-protection techniques—such as generalization or noise addition—before sharing them with each other. However, recent studies have shown that it is possible to deduce the identity of research participants from clinical data that were considered anonymized. DNA sequencing aggravates this problem as the genome is unique to every individual and can be used to predict future ailments for individuals and their blood relatives (such as Alzheimer's or schizophrenia). Such information has the potential to deny jobs and to isolate subjects socially [2]. In the face of these growing concerns, privacy experts are trying to explore alternative approaches to privacy protection. Many of the new strategies are based on cryptography, particularly secure multiparty computations (SMCs). SMC is an attractive approach that allows a set of multiple parties, *S_1_,...,S_m_*, each holding a private fraction of the data to be analyzed, to collectively carry out a computation *f* on the overall dataset, without any party having to reveal their own private raw data. Thus, the goal is to compute *f* efficiently and privately such that no party learns anything aside from the final output of *f*. Note that the output is computed from the private inputs of the different parties, and as such, it may leak some sensitive information about their input. In fact, SMCs focus on the security of the distributed computation method and do not specify which kind of computations can be performed when privacy is of interest. In other words, it does not specify whether the output of a given computation will leak sensitive information or not, it just guarantees that the computation method itself preserves the privacy of the distributed raw data. Techniques from differential privacy have been used (in combination with SMCs) to prevent leakage of sensitive information from the final output. The discussion of these mechanisms is beyond the scope of the study; for more information, readers are referred to the studies by Beimel et al, Nordholt et al, and Papadimitriou et al [3-5].

Despite the mathematical proofs that have been established, demonstrating the ability of the SMC protocols to protect data, they are still not widely used. This may be because knowledge about their capabilities is still relatively small, they tend to have complex solutions that are not accessible without domain knowledge, they require coordinating analyses among the different sites, or they are not efficient in every setting. In fact, one of the main problems with SMC protocols is efficiency. Communication between the different parties is the main factor driving the inefficiency of SMC protocols [6-8]. In almost all existing research in SMC, one of the main goals is to minimize the total number of messages communicated between the different parties and, thus, minimize the performance gap between secure and regular protocols [9]. One of the approaches taken is to relax security and privacy requirements (such as allowing some information leakage) [10].

Our goal with this line of research is to develop usable and efficient SMC applications that meet the needs of the potential end-users and to raise general awareness about SMC as a tool that supports data sharing. Thus, we proposed a divergence from current research efforts. Instead of lowering the security requirements, we proposed to introduce distributed statistical computing (DSC) into the design of secure protocols. Through DSC, we will study the possibility of conducting computations on each of the parties’ sites independently and then combine these (local) computations to form 1 (accurate) estimator for the collective dataset, thus limiting communication to the final step and significantly reducing complexity.

### Contributions

The main contribution of this study is introducing a model for privacy-preserving distributed data mining in which local models are produced separately and SMC is used to aggregate the results privately. The study applies these novel ideas to linear regression and introduces the first secure linear regression model that does model selection and parameter estimation efficiently (all previous secure multiparty algorithms perform parameter estimation only). The paper then presents experiments on real and synthetic datasets to demonstrate the accuracy and efficiency of the algorithm.

The paper is organized as follows: the next section defines our problem formally and introduces the theory behind DSC; the following section demonstrates the effectiveness of our privacy-preserving model through a linear regression application; and finally, the paper is concluded with a discussion of the results and limitations and a proposal for future research directions.

## Methods

### Problem Definition

**Figure 1:**
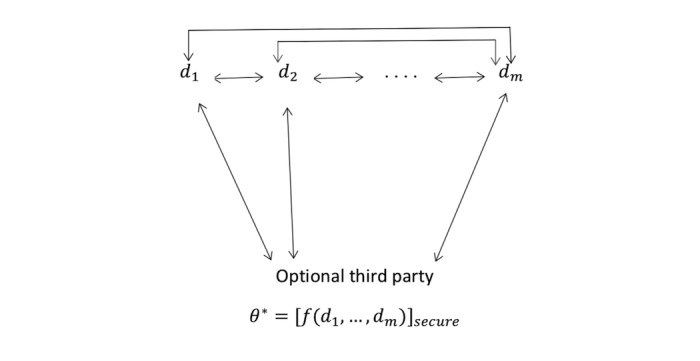
Traditional secure computation approach. Double-sided arrows indicate required communication channels. All communication should be secure and no party (including the third party) should learn anything about other parties' input aside from the final estimation of *θ*. *θ*: population parameter to be estimated; *d_i_*: private dataset owned by site *i* (where *iϵ{1,...,m})*; *f*: a mechanism for the estimation of *θ*; θ*: the output of *f*; *m*: number of sites.

A researcher wants to estimate a population parameter *θ* by running a computation *f* over the private inputs of several remote databases, *d_1_,...,d_m_* while keeping these inputs private (Figure 1); (where *f* (*d_1_,...,d_m_*) is a mechanism for the estimation of *θ*). The goal is to achieve a good approximation θ* of *θ* using as little communication as possible and without any party learning anything about other parties’ input aside from the final output θ* (Figure 1).

Interinstitutional data sharing is generally motivated by multiple scenarios such as (1) increasing results’ accuracy and lowering bias, (2) performing benchmarking studies, or (3) attaining the cohort required for a study. In what follows, we illustrate each with a scenario:

1. *m* hospitals want to collectively study factors that affect the survival rate for breast cancer patients. Running the regression problem on the *m* datasets will provide better properties by increasing sample size and will reduce data bias (such as environmental and location bias). Sharing data in the open may not be easy as medical data are governed by privacy legislations.
2. Hospitals in a given geographical area are interested in calculating the average rate of hospital-acquired bacterial infection (across all the hospitals in the said area) for the purpose of self-evaluation. In this case, the hospitals have an additional incentive against data sharing as it may implicate them negatively.
3. Monogenic diseases are very rare genetic disorders associated with single gene variations observed in few subjects per 1000 to 10,000 individuals. Some are well-characterized such as cystic fibrosis (frequency of disease 1:2000), sickle cell anemia, phenylketonuria (frequency of disease 1:8000), and some primary immunodeficiency diseases [11]. The study of these rare disorders requires the sharing of data across multiple sources or institutions to enable the collection of more cases for analysis and thus increase the statistical power of the study.

Many protocols have been developed for the above problem in the area of SMC. The most efficient protocols are based on secret sharing [12], oblivious transfer [13], garbled circuits [14], or homomorphic encryption [15]. In addition, there are several hybrid constructions that combine these various models [10]. These protocols have robust mathematical proofs that demonstrate their ability to protect privacy under different assumptions of parties’ honesty [10]. However, they mostly involve heavy communication (extensive message passing) between the different concerned parties [9]. To minimize the communication load and decrease the performance gap between secure and regular protocols, researchers tried to relax security and privacy requirements such as relaxing the number and power of dishonest parties or allowing some form of information leakage [10], others use noise addition to intermediate and final results to preserve privacy [16]. In this study, we proposed a change in the methodology by introducing distributed statistical learning into the design of secure computations.

### Statistical Learning With Big Data

#### Overview

**Figure 2:**
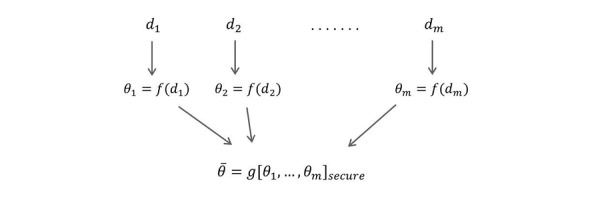
The split and merge approach. The one-sided arrows indicate message passing. All communication should be secure and no party (including the optional third party) should learn anything about other parties' input aside from the final estimation of *θ*. *θ*: population parameter to be estimated; di: private dataset owned by site *i* (where *iϵ{1,...,m})*; *f*: a mechanism for the estimation of *θ*; *θ_i_*: the output of *f* applied to *d_i_*; *g*: a mechanism for combining local estimates; *θ^#^*: the output of *g*; *m*: number of sites.

A common approach in statistical learning with big data is to split the data (along observations) into multiple subsets. Each subset conducts the required computation completely independently. The final result is then obtained by combining these local computations. Thus, communication (and sharing of information) is reduced to the final step only. This will significantly reduce the complexity and provide simpler algorithms. The problem is illustrated in Figure 2 and explained below.

A researcher is interested in estimating a population parameter *θ* from a sample database *D* with *N* records and *p* attributes. Traditionally, *θ* is estimated from the whole dataset *D* as: θ*=f (*D*) (referred to as the centralized estimator), where *f* is a mechanism for the estimation of *θ*. In this *split and merge* statistical learning approach, the database *D* is split equally among *m* sites as *d_1_,...,d_m_*. The number of records in the resulting databases is denoted by *n=N/m*. Each site *i* performs the estimation of *θ* on its local dataset as: *θ_i_=f* (*d_i_*), then the final estimate is obtained by combining the local estimates: *θ^#^=g* (*θ_1_,...,θ_m_*).

The *N* observations are assumed to be independent and identically distributed. They are evenly and randomly allocated along the *m* different sites.

McDonald et al, who advocated for this *split and merge method* in [17], claim that, given the stated assumptions, it provides a good balance between accuracy and efficiency. As for merging strategies, few were considered in the literature, the most common ones being averaging [18] and median [19].

#### Relevant Theory

The *split and merge* strategy is simple to execute and is communication-efficient. Splitting is always done along observations (rather than attributes), and each site performs the estimation on its local dataset. Averaging of the *m* sites estimates is the simplest and most popular strategy. In what follows, we review the available literature while trying to answer the following questions:

- What is the error of the averaged estimator versus the centralized one?
- What affects the optimality of the averaged estimator?
- How many sites to include in a given study? And how many samples to include from these sites?

The accuracy of averaging depends on the relationship between the number of observations (*N*), the number of sites (*m*), and the number of parameters (*p*). As a general insight, averaging provides estimates that are as accurate as the centralized solution when there are many observations per parameter on each local machine [20]. In fact, in [21], the authors proved that when the number of records per site is large, (large *n=N/m*), the mean square error (MSE) of the average estimator (ie, E||*θ^#^-θ* ||^2^) is the same as the MSE of the centralized one (ie, E||θ*-θ ||^2^). In [18], Rosenblatt and Nadler proved that the averaged estimator and the centralized one are first order-equivalent, they proved that the leading error term of (*θ^#^-θ*) and (θ*-θ) converge to the same limit at the same rate; however, some accuracy loss is exhibited in higher-order terms for nonlinear models (the second-order term is *m* [number of sites] times larger than the first-order one). The interpretation as given in [18] is that first-order terms generally capture variance, which is reduced by averaging, whereas the second-order term captures bias which is not reduced by averaging. Thus, the old problem of balancing variance and bias comes to light in nonlinear models (where the second-order term can be nonnegligible). Approaches toward this problem can be found in [20-22]. Going further, Rosenblatt and Nadler presented an extensive analysis of the error of the averaging estimator by considering different practical situations [18]:

- For situations where *p* is fixed and *n* is large, they proved that the averaged estimator, *θ^#^*, is asymptotically equivalent to the centralized one, θ*.
- For small and medium *n*, parallelization incurs a non-negligible error for nonlinear models.
- For situations where *N* is fixed, they showed that averaging performs well for small values of *m* and *p*.

The authors presented the exact expression of the estimator errors in all situations and confirmed the results through a series of experiments.

In [20], the authors proved that (for low-dimensional data) it is enough to have a smaller number of sites than local observations (*m≤n* or, equivalently, *m≤N^1/2^*) to guarantee an MSE that decays at a considerably better rate than the centralized approach. They also showed that when *N* is fixed, the MSE of the averaged estimator increases polynomially with the number of sites *m*, thus echoing previous theoretical results.

Battey et al specified in [23] an exact formulation of the requirements on *m, N,* and *p*, for linear models. They proved that when *p<log N* and *m≤Np/* (*log N*)*^2^*, then the loss incurred by the averaging method is negligible compared with the statistical error incurred by the central one.

As a general insight into the questions raised above and to summarize the above results, we say that when the number of samples per site *n* is large, bigger than the number of features and bigger than the number of sites (*n>p* and *n > m*) then the averaged machine-wise estimates are as accurate as the centralized estimates. However, when the number of samples per site, *n,* is small and the model is highly nonlinear, the error can be non-negligible. The nice results do not extend to high-dimensional data. When few observations are available per parameter per site (*n<p*), in these cases the accuracy loss increases linearly with the number of sites *m*. Some researchers, such as [24], resorted to schemes other than averaging to obtain well-behaved estimators in specific cases, whereas others showed moderate accuracy loss for averaging in specific cases [18,25,26].

#### Assumptions and Considerations

The assumptions across the DSC literature are that (1) the *N=mn* observations are independent and identically distributed according to a distribution *P* and that (2) they are evenly and randomly allocated along the *m* different sites.

An equivalent assumption to (1), that applies to our SMC scenarios, is that of *m* independent sites having observations that are independent and identically distributed according to the same distribution *P*.

The (second) assumption of evenness can be easily circumvented by pre-setting the number of samples to consider from each site. However, that is not needed, as the theoretical results presented in the previous section would still apply provided that every site satisfies the required assumptions (ie, the assumptions are true for each *n_i_*, the number of observations for site *i*).

However, the first assumption is not always realistic and can be overly restrictive for some applications. For instance, if the data are already owned and collected by the different sites, then it may exhibit systematic differences across these sites. For example, if 2 hospitals are considering an analysis involving their cancer patients and if 1 of the hospitals is located in a heavily polluted area whereas the other is not, then the distribution of the local population from which the sites’ data are sampled could have significant differences. Pooling the data and redistributing them randomly along the different sites may not be realistic or feasible as the data may be big or private [19].

Going back to the question of the number of sites to include in a study when *p* is fixed, the authors in [18] distinguished between 2 scenarios *N* fixed or *n* fixed. Fixed *N* captures the case of limited data availability or limited computational power whereas fixed *n* captures the case of storage restriction or data availability per site. For the SMC problem, where data are already owned by the different sites, fixed *n* represents the case of a given number of institutions (sites) wanting to run an analysis on their collective data (with *n* being the minimal number of samples across sites). Fixed *N* represents the case of a researcher with a requirement on cohort size and is assumed to be able to include as many sites as required to attain the cohort (with each site having at least *N/m* records). The authors in [18] presented an analysis of (1) the minimal number of sites to attain a desired accuracy in the fixed *n* scenario and (2) the maximal number of sites to attain a desired accuracy in the fixed *N* scenario. The objective is to guarantee an MSE that is lower than a preset value as follows: min {*m*; MSE(*m*)≤ϵ with fixed *p* and *n* } and max {*m*; MSE(*m*)≤ϵ with fixed *p* and *N* }. For example, in the fixed *N* scenario with *p*=10^3^ if *N*=10^6^, *m* should be ≤*899* to guarantee an MSE under 0.1 [18].

We distinguish between these 2 strategies when analyzing the performance of our algorithm.

#### Multiestimators

In many applications, certain inferences require 2 or more estimations. For example, inference for regression typically requires 2 components—feature selection and parameter estimation. When conducting feature selection, the median probability model has been recommended [27]; it consists of all the features that are selected by the majority of the subsets. According to [27], the median model produces the best approximation under some simplifying assumptions, in that it has the highest probability of being equal to the optimal model. Averaging is not recommended as it can lead to a bigger number of nonzero coefficients and, thus, to an inflation in the number of selected features as opposed to median. The median selection model is also less influenced by the heavy presence of outliers when compared with the central selection model, as the effect of the outliers will be waned down over multiple subsets (only a fraction of the subsets will contain a sizable fraction of the outliers) [28].

In [28], the authors present a distributed linear regression algorithm that combines median model for feature selection and simple averaging for parameter estimation. The authors proved that for low-dimensional data, when the features are independent and the number of sites is well chosen (number of sites *m* chosen so that *m<n*) or when features are correlated and following elliptical distributions (noting that real-world data commonly follow elliptical distributions), the distributed model provides accurate estimates. In fact, they showed that their algorithm can achieve better accuracy in terms of feature selection than the centralized one, which results in a better MSE in general. The authors performed extensive experiments (with *p<N*) that echoed their theoretical results. However, their choice of number of sites versus sample size always satisfied Zhang et al’s condition [20], (*m≤N^1/2^*).

In the next section, we demonstrate the effectiveness of the privacy-preserving model through a linear regression application. We restrict our application to models with the best theoretical results, that is, linear models with more records than features in every site and with high number of records relative to the number of sites (*n>p* and *n>m*).

## Results

### Application: Secure Linear Regression

We introduce the classical setting of a linear regression problem. Let *X*={*x_i,j_* } be an *N×p* matrix of features and *Y*=(*y_1_,...,y_N_*)*^T^* a corresponding *N* ×1 response vector, where *N* is the number of samples and *p* is the number of features. Linear regression consists of modeling the relationship between the set of features (also known as independent variables) and the response variable. It assumes that the relationship between the response variable and the independent variables is linear. Fitting a linear regression model consists of feature selection and parameter estimation [29]. Feature selection is the process of constructing a model that includes all relevant predicting variables. In other words, it is the process of determining the subset of features that best predicts the outcome variable, *Y*, whereas the parameter estimation consists of finding the linear model parameters *β* where *Y=Xβ+ϵ* [29].

Despite the simplicity of linear regression, it is widely used in various biomedical applications [30]. Although physical and biological processes are inherently nonlinear, linear approximations have been successfully used for centuries to explain phenomena in physics and biology [30] as they present a number of advantages compared with more complex models. Parameters of linear models are usually easy to estimate, the linear models are easy to interpret (coefficient signs and values are indicative for the contribution of the different variables), and many tools have been developed to evaluate the statistical significance of linear models. Linear models are also well-suited for high-dimensional data and are used for association studies such as Genome Wide Association Studies.

### Previous Work in Secure Linear Regression

As linear regression is one of the most commonly used statistical tools in data analysis, there are many attempts in the literature at obtaining secure linear regression protocols over distributed databases. Many of these protocols do not offer adequate privacy guarantees [31,32] as they share intermediate results or rely on a trusted third party to handle these intermediate results [33]. In [34], El Emam et al provide some scenarios where privacy can be breached by sharing intermediate aggregate results (refer to [35] for a decomposition of available secure regressions based on privacy and accuracy). The first linear regression algorithm with cryptographic security was developed by Hall et al [15]; it makes heavy usage of SMCs, particularly secure matrix multiplication protocol. The study reported 2 days for solving a linear regression problem of 51k rows and 22 features [15]. In [36], a solution based on homomorphic encryption and garbled circuits is presented. The solution uses 2 noncolluding semihonest third parties. The problem with the approach is that usage of garbled circuits imposed many rounds of interactions and is thus heavy on communication. In a more recent experiment [33], the authors report 8.75 hours for 10^8^ records with 20 features and 270 MB of communication. In 2015, Cock et al, presented a method to calculate the parameters of the linear regression by computing *β*=(*X^T^X*)*^-1^X^T^Y* [37]. The algorithm computes β by running Beaver’s matrix multiplication protocol many times [38]. Beaver’s protocol computes securely, with the help of a trusted initializer, the product of matrices shared by different parties in a way that the result remains shared by the different parties. The theoretical complexity of the algorithm is *O* (*Np^2^*); however, the protocol is heavy on communication. In fact, the matrix multiplication protocol requires each party to send 2 matrices to every other party (of size, *p^2^*), such protocol is repeated, *O* (*k*), where *k* is the maximal number of bits required to represent the largest integer. However, their algorithm performs better than all previous secure linear regression algorithms [37]. Experiments done by the authors themselves indicated a capacity to handle over 4 million records with 16 features in a range of 3 hours (to provide some perspective, our algorithm requires less than 3 min for the same dataset and same settings and for both feature selection and parameter estimation).

It is very important to note that all cited secure regression algorithms do not perform feature selection. In other words, they use the supplied features set to predict the model [35]. As our algorithm does both, we opted to compare our algorithm with the central version (where data are on the clear).

### Our Algorithm

The goal of distributed statistical learning algorithms is to scale up computations by distributing the data over multiple machines. The underlying assumption is that all data are owned by the same organization. Thus, sharing of intermediate and local results between the different machines is allowed.

In our setting, the dataset (*X, Y*) is owned by *m≥* 2 data holders (or sites) *S_1_,...,S_m_* and the different sites are interested in cooperatively performing linear regression on the union of their datasets; however, they are not willing or able to share their data. Only the final result of the computation should be revealed to all parties. In line with the DSC theory, we assume that all the samples in all sites are independent and identically distributed (randomly drawn from the same [population] distribution). Moreover, if *n_min_* is the smallest number of local observations across the different sites, (to guarantee the nice DSC results) we require that the number of features and number of sites are both smaller than *n_min_*, that is, we require that *n_min_≫p* and *n_min_≫m*.

Formally, the data (*X,Y*) are divided horizontally into *m* subsets {(*X^1^*,*Y^1^*);...;(*X^m^*, *Y^m^*)}, with *X^i^*=(*X*_1_*^i^,...,X^i^_p_*) the *n_i_* ×*p* feature matrix for subset *i* (where *X^i^_j_* is an *n_i_×1* matrix) and *Y^i^*=(*y_1_^i^,...,y^i^*_*n_i_*_)^*T*^ the corresponding *n_i_×1* response vector. The algorithm then executes the following 2 steps:

1. Each site calculates its local feature selection vector privately, and the local vectors are aggregated securely using a secure median algorithm (in other words, the parties jointly perform the median on their data and obtain the result), without any party revealing any information about their selected features (aside from what can be deduced from the final median output).
2. Next, each site uses the shared selected features to calculate the model parameters locally. These local parameters are then securely averaged using a secure average protocol. Our algorithm is presented in Multimedia Appendix 1. In the algorithm, the secure sum and secure median protocols are based on Paillier homomorphic encryption; however, other secure protocols can be used instead.

### Experiments

We evaluated our secure multiparty linear regression algorithm (SMA) by implementing it and analyzing the results using real and synthetic datasets. The real datasets are used to test the accuracy of the algorithm whereas the synthetic datasets are used to analyze its performance. To analyze the accuracy of the algorithm, we needed real datasets that originated from multiple different sources (different data owners). The sources’ IDs had to be included to inform the actual data division along the different sites. For the synthetic experiment, data were generated and randomly allocated along the different sites, as the purpose was solely to evaluate the efficiency of the algorithm for various numbers of records and features. We used Python3 as our programming language, which we augmented with the Scikit-learn, numpy, pandas, gmpy2, and phe libraries for functionality such as socket programming, homomorphic encryption, and for dealing with negative and real floating-point arithmetic. We built our system on top of 10 Linux machines with Intel Core i5-4570 CPU, 3.20GHz processor, and 8GB RAM, 4 cores each. To increase the number of possible sites to 20, we installed 2 Linux virtual machines on each machine with 4 GB memory each (note that the Paillier encryption library handles real-number values with arbitrarily high precision).

To test our SMA, we compared its performance with the central algorithm (CA). The CA performs linear regression on 1 machine using the same approach as the SMA (ie, it uses lasso for feature selection and linear least squares method for parameter calculation [39]). We opted not to test the accuracy or the efficiency of our SMA algorithm against existing secure linear regression algorithms as none of the existing algorithms perform model selection.

#### Real Datasets

To test the accuracy of our algorithm, we collected real datasets that include information about the original collection site. Then, we treated each site as an independent data owner. We succeeded in finding 4 real datasets: 3 public datasets contained within the University of California Irvine repository (student performance in Portuguese, student performance in Math, and automobile fuel consumption data) and 1 from Cerner clinical database (the Diabetes dataset, where the number of sites included was varied between 3, 6, and 12, and the weight variable was excluded in some experiments because of excessive missing values). The datasets are presented in detail in Multimedia Appendix 2.

In the experiments on real datasets, we randomly divided the datasets into 0.7 training set and 0.3 testing set. A regression model was trained based on the training set and used to predict the outcome variable in the testing set. The average of the square prediction error was used to evaluate the model (MSE). The experiments were repeated 50 times each. Table 1 summarizes the results; as evident from the results, our method does not incur significant loss in accuracy.

**Table 1:** Performance results for the 4 datasets used.

Dataset	Mean (SD)	*m* ^a^	*p* ^b^	*N* (values of *n*)^c^	MSE^d^		*R* ^2^		MSE ratio^e^
					CA^f^	SMA^g^	CA	SMA	
Student performance in Portuguese^h^	11.91 (3.23)	2	30	649 (423, 226)	3.364	3.417	0.68	0.675	0.984
Student performance in Math^h^	10.41 (4.58)	2	30	395 (349, 46)	7.554	7.719	0.627	0.62	0.978
AutoMPG^i^	23.45 (7.80)	3	6	392 (245, 68, 79)	13.56	17.563	0.778	0.711	0.77
Diabetes (with weight)^j^	4.848 (3.11)	3	39	267 (129, 72, 66)	8.801	8.59	0.09	0.108	1.025
Diabetes (with weight)^j^	4.41 (3.02)	6	39	456 (68, 130, 57, 73, 55, 73)	7.443	7.733	0.19	0.157	0.962
Diabetes (without weight)^j^	4.39 (3.01)	3	38	8567 (2478, 3936, 2153)	5.558	5.612	0.309	0.303	0.99
Diabetes (without weight)^j^	4.42 (3.00)	6	38	13626 (2478, 3936, 1480, 2153, 2108, 1471)	5.708	5.798	0.345	0.335	0.984
Diabetes (without weight)^j^	4.39 (2.97)	12	38	21205 (2478, 3936, 1480, 2153, 2108, 1471, 1160, 1323, 1524, 1425, 1024, 1122)	5.705	5.87	0.345	0.336	0.971

^a^*m*: number of sites.

^b^*p*: number of features.

^c^*N* (values of *n*): total number of records and their division along different sites.

^d^MSE: mean square error.

^e^MSE ratio=MSE CA/MSE SMA.

^f^CA: central algorithm.

^g^SMA: secure multiparty linear regression algorithm.

^h^Outcome variable: grade out of 20.

^i^Outcome variable: fuel consumption (miles per gallon).

^j^Outcome variable: length of stay (days).

#### Synthetic Dataset

**Figure 3:**
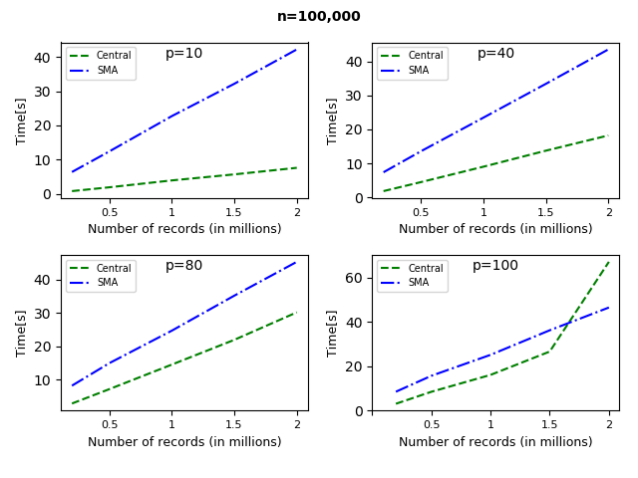
Time performance for central algorithm versus secure multiparty linear regression algorithm (SMA) with 100,000 records per site and varying feature set. As the number of sites increases, the number of records also increases. For small datasets, the time taken by SMA is more than that taken by the central algorithm (CA). This is due to the encrypted communication required by the algorithm. As the number of records and features increases, the time taken by the CA increases rapidly (at 1,500,000 records and 100 features). *n*: number of records per site; *p*: number of features.

**Figure 4:**
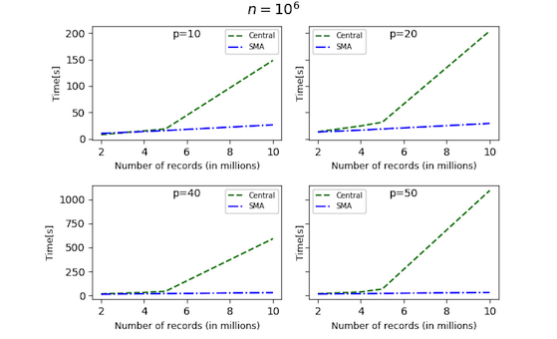
Time performance for central algorithm versus secure multiparty linear regression algorithm (SMA) with 1,000,000 records per site and varying feature set. The time taken by the central algorithm (CA) is greater than that taken by the SMA. For 10 million records, the SMA algorithm takes almost 30 seconds, whereas the CA takes around 18 minutes. *n*: number of records per site; *p*: number of features.

**Figure 5:**
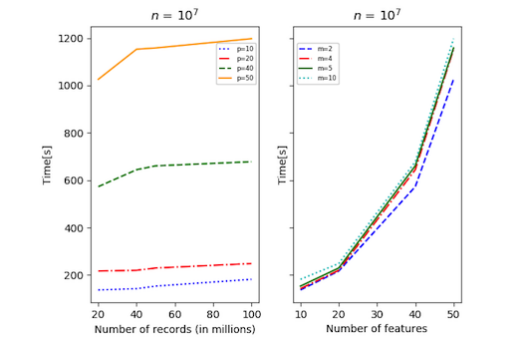
Time performance for secure multiparty linear regression algorithm (SMA) with 10 million records per site and varying feature set. The panel on the left shows the time as a function of the number of features, while the panel on the right shows the time as a function of the number of sites. Note that for *N*=100 million and *p*=50 features, SMA required 20 minutes for execution. *m*: number of sites; *n*: number of records per site; *p*: number of features.

**Figure 6:**
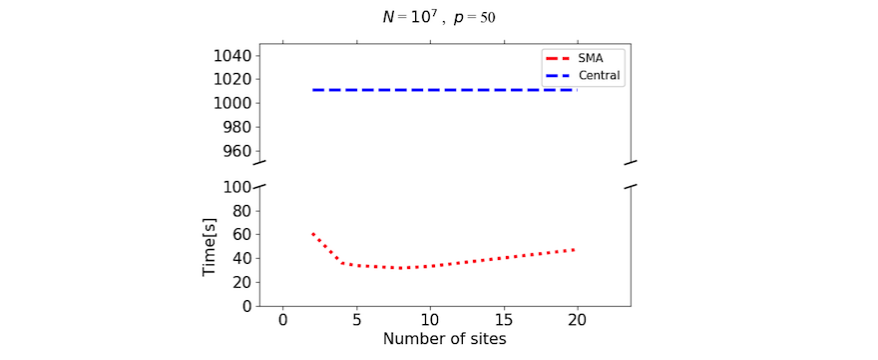
Time performance for central algorithm versus secure multiparty linear regression algorithm (SMA) with total number of records (*N*)=10 million, and features (*p*)=50. For SMA, the records are divided among a varying number of sites (2 to 20). The time taken by the central algorithm (CA) is constant. For SMA, time decreases with the increase in the number of sites, until it reaches *m*=20 (or *n*=50,000). At that point, the communication cost increases and the computation time starts to go up.

Using synthetic data, we performed a scalability analysis to evaluate the time performance of the proposed solution as the data size and the number of parties increase. The synthetic datasets were generated in Python using sklearn.datasets.make_regression. The number of records was varied between 10^5^ and 10^8^, the number of features between 2 and 50, and the number of sites between 2 and 20. The records were always divided equally between the sites. We distinguished between 2 testing strategies: *n* fixed (Figures 3-5) and *N* fixed (Figure 6). The algorithm was compared with the CA (where data are shared in the clear) as there exists no other secure linear regression algorithm that performs model selection.

For the fixed *n* strategy (with, *p≪n*), Figures 3 and 4 show the time performance of CA versus SMA as a function of the sample size *N*. Note that *m=N/n* is also growing (*m* ∈ [2,20] in Figure 3, and *m* ∈ [2,10] in Figure 4). As seen from the figures, for large *n* and *p*, SMA is scalable, and the security overhead does not affect its performance significantly. Figure 5 shows the time performance of SMA (with *n*=10 million) as a function of (1) *N* (left side) and (2) *p* (right side). Note that *N* varies between 20 million and 100 million and that the time performance for *N*=100 million and *p*=50 features is under 20 min.

For the fixed *N* strategy (with *p≪N*) Figure 6 shows the time performance of SMA as a function of the number of sites (with *p*=50). Note that the time taken by the CA is constant whereas for the SMA, as the number of sites increases, the time taken by the algorithm decreases. It is important to note that when the number of records per site becomes very small, the communication cost increases, driving the overall computation time with it.

## Discussion

This study introduced a model for privacy-preserving distributed data mining in which local models are produced separately and SMC is used to aggregate the results privately. Theoretical results from statistical theory were used to design the first secure multiparty linear regression model that does model selection and parameter estimation.

In general, theoretical results from statistical computing say that the averaged local estimates are as accurate as the centralized estimates when *n>p* and *m<n*. In line with the theoretical results, we conducted computations on the distributed sites independently and then combined the results securely to form 1 estimator for the collective dataset. Experiments were conducted with *n ≫ p* and *m < n* and they showed the accuracy (using 4 real datasets) and efficiency (using synthetic data) of the algorithm.

The experiments on synthetic data showed very good time performance. When *n* is fixed, as *N* increases, the time taken by the CA increases at a much faster rate than SMA. For big *N* (10^8^), the algorithm does model selection, and parameter estimation in under 20 min (the algorithm of [36] does only parameter estimation in the range of 8 hours).

Much of the existing theoretical work in DSC assumes a uniform and random distribution of samples across the different sites or that the *m* independent sites have *n* observations each that are independent and identically distributed according to the same distribution *P*.

This assumption certainly facilitates the mathematical analysis but may not be realistic for some applications. In the SMC applications, data are collected and owned by the different sites and may thus have systematic differences across these different sites, in which case, the assumption could be overly restrictive. Redistributing the samples randomly across the different sites is not an option due to data privacy issues. However, it is worth noting that our experiments on real data (although limited due to lack of access to real data) showed high accuracy compared with the central case. In the future, we intend to relax these assumptions and study their theoretical effect on the accuracy of the results.

Another limitation is the assumption of horizontal distribution among the different sites which should be generalized to vertical divisions (or both).

Moreover, the study demonstrated the theoretical results using a linear model. We plan to extend our results to other estimators such as ridge regression and logistic regression.

## Appendix

Multimedia Appendix 1 Secure multiparty algorithm.

Multimedia Appendix 2 Real datasets.

## Abbreviations

CA: central algorithm
DSC: distributed statistical computing
MSE: mean square error
SMA: secure multiparty linear regression algorithm
SMC: secure multiparty computation
