# Supplementary material for: Privacy-Preserving Analysis of Distributed Biomedical Data: Designing Efficient and Secure Multiparty Computations Using Distributed Statistical Learning Theory
Source: JMIR Med Inform. 2019 Apr 29;7(2):e12702. doi: 10.2196/12702 (PMC6658266; doi:10.2196/12702)
Supplement: Multimedia Appendix 1 [file medinform_v7i2e12702_app1.pdf]

# MULTIMEDIA APPENDIX 1

In our setting, we assume that the dataset $(X, Y)$ is owned by $m \geq 2$ data holders (or sites) $S_1, \dots, S_m$ and the different sites are interested in cooperatively performing linear regression on the union of their datasets, however they are not willing to share their data. Only the final result of the computation should be revealed to all parties.

Formally, the data $(X, Y)$ is divided horizontally into $m$ subsets $\{(X^1, Y^1); \dots; (X^m, Y^m)\}$, with $X^i = (X_1^i, \dots, X_p^i)$ the $n_i \times p$ feature matrix for subset $i$ (where $X_j^i$ is an $n_i \times 1$ matrix) and $Y^i = (y_1^i, \dots, y_{n_i}^i)^T$ the corresponding $n_i \times 1$ response vector. The algorithm then executes the following two steps:

1. Each site calculates their local feature selection vector privately, and the local vectors are aggregated securely using a secure median protocol.
2. Next, each site uses the shared selected features to calculate the model parameters locally. These local parameters are then securely averaged using a secure average protocol.

   An example for our method is provided in detail in Figure 1 and Figure 2 below. In this example the secure sum and secure median protocols are based on Paillier homomorphic encryption, however that other secure protocols can be used instead. The algorithm was first presented in [1]

1. A semi-trusted third party generates the keys for the Paillier cryptosystem and propagates the public key to all parties
2. Each party calculates its local feature selection vector via Lasso, the overall feature selection vector is then calculated using the secure median protocol. The secure median protocol retains all features that have an overall inclusion probability greater than ½. The calculated median vector is then propagated to the different parties without leaking any information about individual feature selection vectors. Thus, if $\gamma^i = \{\gamma_1^i, \dots, \gamma_p^i\}$ is the feature selection vector for site $i$, (with $\gamma_j^i = 1$ if feature $j$ is included and 0 otherwise), then the overall vector is obtained by: $\gamma = \{median\{\gamma_1^1, \dots, \gamma_1^m\}, \dots, median\{\gamma_p^1, \dots, \gamma_p^m\}\}$

3. After receiving the overall feature selection vector, each party calculates the coefficients of the selected features separately. The encrypted average of these features is then securely computed using Paillier encryption: thus, if $\beta^i = \{\beta_1^i, \dots, \beta_p^i\}$ is the feature coefficient vector for site $i$, then $\beta = Secure\{Average\{\beta_1^1, \dots, \beta_1^m\}, \dots, Average\{\beta_p^1, \dots, \beta_p^m\}$: each site $i$ calculates $Enc(\frac{\beta^i}{m})$, then all sites calculate $Enc\left(\sum_{i=1}^{m} \frac{\beta^i}{m}\right) = \prod_{i=1}^{m} Enc(\frac{\beta^i}{m})$ sequentially. The encrypted result is sent to the third party which decrypts and propagates the estimated feature coefficients.

**Figure 1.** Steps for the secure linear regression algorithm

It is important to note that the algorithm can be slightly changed to operate without the need of a third party. In such case, a threshold Paillier cryptosystem can replace the third party. For more information, readers are referred to [1].

**Initialization**

1: $\theta = \{\frac{1}{2}\}^p$

**Each party $i$**

2: Generates a random positive integer $x_i$ # *denote by* $x = \prod_{i=1}^{k} x_i$

**Each party calculates**

3: $w^i = (\gamma^i - \theta)$

4: $Enc(w^i)$

**All parties calculate**

5: $Enc(w) = \sum_{i=1}^{k} Enc(w^i)$ # *note if* $w$ *is positive at position* $j$ $(w[j] > 0)$ *this implies that the majority of sites had 1 at the* $j$ *position in their feature inclusion model*

**Sequentially the parties calculate**

6: $Enc(wx) = \{Enc(w[1])^x, \dots, Enc(w[p])^x\}$ # *party1 calculates* $Enc(wx_1) = \{Enc(w[1])^{x_1}, \dots, Enc(w[p])^{x_1}\}$ *and sends it to party2, party 2 calculates* $Enc(wx_1x_2) = \{Enc(wx_1)^{x_2}, \dots, Enc(wx_1)^{x_2}\}$ *and so on.*

7: $Enc(wx)$ is sent to third party

**Third party**

8: Decrypts $Enc(wx)$

9: Propagates $wx$

**Each site**

10: Calculate γ as follows $\begin{cases} \gamma[\mathrm{j}] = 1 \text{ if } \mathrm{W}[\mathrm{j}] > 0 \\ \gamma[\mathrm{j}] = 0 \text{ otherwise} \end{cases}$

**Figure 2.** The secure median algorithm

The complexity of the algorithm is $np^2$ and total communication required is 2 messages per party, where each message is of size $p$ integers. This is a big step forward in terms of the communication required and it is due to the fact that model selection and regression parameters are calculated locally. And that communication is required to aggregate the local results at 2 different instances in the program execution.

References


1. Dankar FK, Boughorbel S, Badji R. Using Robust Estimation Theory to Design Efficient Secure Multiparty Linear Regression. In EDBT/ICDT Workshops; 2016 March 15-18; Bordaux, FR: 2016.
