# Supplementary material for: Privacy-Preserving Analysis of Distributed Biomedical Data: Designing Efficient and Secure Multiparty Computations Using Distributed Statistical Learning Theory
Source: JMIR Med Inform. 2019 Apr 29;7(2):e12702. doi: 10.2196/12702 (PMC6658266; doi:10.2196/12702)
Supplement: Multimedia Appendix 2 [file medinform_v7i2e12702_app2.pdf]

MULTIMEDIA APPENDIX 2

To test the accuracy of our algorithm we needed real datasets that include information about the original collection site (in other words, the sites where the data originates from should be known) and every site is treated as an independent data owner. We were able to find 4 real datasets, 3 public datasets contained within the UCI repository and one from Cerner clinical database:

Student Performance Data Set (Portuguese performance and Math performance):

These two datasets are related to student performance in two Portuguese schools for two subjects Portuguese language and Mathematics. The total number of students is 649 with 30 attributes. The data attributes include first term grades, demographics, social and school related features. The target attribute is the final year grade (out of 20). Both datasets were divided into 2 sites based on the school name.

Auto-Mpg Data

This dataset is related to car fuel consumption in miles per gallon. It contains 392 records with 9 features including car origin (manufacturer), cylinders, weight, acceleration, and miles per gallon. The variable to be predicted is mile per gallon (mpg). The dataset is divided into 3 sites according to the 'origin'. The number of records in the sites were 245, 68 and 79 respectively

Diabetes Data set

This is a clinical diabetic dataset with 101,767 records and 41 features, including hospital_id, race, gender, age, weight, admission type, glucose measurements (at different times), insulin doses, A1C results, and diagnosis. The dataset was divided into multiple sites based on the 'hospital_id'. We performed several experiments on this dataset by varying the number of sites used (3, 6 or 12 sites) and by including or excluding the 'weight' feature (note that the 'weight' is an important predictor, however it is missing from 90% of the records). The response variable is 'times in hospital', that is the length of stay in hospital with a range of 1 to 14 days.
